# Protein modification by a eukaryotic-like ubiquitin-related modifier in the hyperthermophilic archaeon *Saccharolobus islandicus*

**DOI:** 10.1128/msystems.00580-25

**Published:** 2025-10-20

**Authors:** Jingjing Cao, Daijiang Xiong, Xiaowei Zheng, Wanjuan Yuan, Li Huang

**Affiliations:** 1 Faculty of Life Science and Technology, Kunming University of Science and Technology 47910, Kunming, Yunnan, China; 2 State Key Laboratory of Microbial Resources, Institute of Microbiology, Chinese Academy of Sciences 85387, Beijing, China; 3 University of Chinese Academy of Sciences 74519, Beijing, China; 4 Key Laboratory of Urban Environment and Health, Institute of Urban Environment, Xiamen, Fujian, China; 5 Key Laboratory of Microbial Pathogenesis and Interventions of Fujian Province University, the Key Laboratory of Innate Immune Biology of Fujian Province, Biomedical Research Center of South China, College of Life Sciences, Fujian Normal University, Fuzhou, China; 6 Southern Marine Science and Engineering Guangdong Laboratory (Guangzhou), Nansha, Guangzhou, China; University of Minnesota Twin Cities, Minneapolis, Minnesota, USA

**Keywords:** ubiquitin-like Urm1, urmylation sites, knock-down mutant, *Saccharolobus islandicus*, Archaea

## Abstract

Although homologs of the eukaryotic Urm1 (ubiquitin-related modifier-1) have been characterized in Archaea, the *bona fide* substrates and roles of the archaeal Urm1 remain poorly understood. Here, we report a proteomic analysis of Urm1 modification in *Saccharolobus islandicus* using a highly efficient method, which involves the introduction of an H81R substitution into Urm1 encoded by the strain, treatment of the strain with the proteasome inhibitor bortezomib, and affinity enrichment of urmylated peptides with an anti-K-ε-Gly-Gly antibody following peptide fractionation. Extensive protein urmylation was observed, with a total of 783 Urm1 conjugation sites, mapped to 330 proteins, identified in the cell. Among the seven lysine residues in Urm1, six were sites of modification, of which K7 and K37 were preferentially modified. Treatment with the proteasome inhibitor bortezomib resulted in K37-linked chains being the sole major modification species, suggesting that K37 linkage served as a primary trigger of proteasomal degradation. The modified proteins were involved in a number of cellular processes, such as cell division, chromosomal organization, DNA replication, translation, proteasomal protein degradation, and sulfur relay. Protein urmylation was dynamic and influenced by growth conditions and stress treatments. Attempts to delete *urm1* were unsuccessful, pointing to the essentiality of the gene. The knockdown of *urm1* resulted in substantial growth delay, during which a drastic reduction in cellular concentration of cell division proteins (CdvB, CdvB1, CdvB2) occurred. Our results shed significant light on the landscape and potential roles of protein urmylation in Archaea.

**IMPORTANCE:**

Although protein urmylation has been documented in Archaea for over a decade, the authentic substrates and functional roles of archaeal Urm1 remain largely unknown. In this study, we generated the largest Urm1 modification data set in Archaea through an efficient *in vivo* approach and investigated its physiological functions in *Saccharolobus islandicus*. Extensive protein urmylation was observed, with modified proteins implicated in key cellular processes such as cell division, chromosomal organization, translation, and proteasomal degradation. Our findings challenge the prevailing notion that Urm1 homologs modify only a limited number of substrates. Six out of seven lysine residues in Urm1 were modified, suggesting the presence of diverse Urm1 chain structures. These results provide cellular evidence supporting the hypothesis that eukaryotic Ub/Ubl systems have an archaeal origin. We also explored how various factors affect global protein urmylation and examined the impact of *urm1* knockdown on cell growth.

## INTRODUCTION

Ubiquitin (Ub) and the related ubiquitin-like proteins (Ubls) are capable of covalently attaching to a diverse array of cellular protein targets, orchestrating a wide variety of regulatory processes in eukaryotic cells (1). Ubiquitin, a conserved 76-amino-acid-long polypeptide, adopts a well-defined β-grasp fold (2). Thousands of substrates can be ubiquitinated via isopeptide bonds by a cascade of E1, E2, and E3 enzymes, a process that is reversed by specific deubiquitinases (3–5). One of the most studied roles of ubiquitination is its pivotal function in proteasome-mediated proteolysis (6, 7). Ubiquitin can be attached onto other proteins as a monomer or as a poly-ubiquitin chain of diverse structural topologies. Notably, K48-linked chains are the most common signal for proteasomal degradation, and K63-linked chains are abundant upon stress (8–10). In addition to its role in protein turnover, ubiquitin functions as a post-translational signal to regulate various cellular processes and pathways, including DNA damage response, cell cycle progression, protein trafficking, apoptosis, and innate immunity (1, 11–14). Moreover, free ubiquitin in the cell is crucial for cell survival, and reduced levels of free ubiquitin compromise the viability of both yeast and mice (15, 16).

The eukaryotic Urm1 (ubiquitin-related modifier-1) protein, a member of Ubls, which exhibits both structural and amino acid sequence homology to the prokaryotic sulfur-transfer proteins ThiS and MoaD, has been proposed as a candidate for the evolutionary “missing link” between the eukaryotic Ub/Ubl family and the ancestral prokaryotic sulfur-transfer proteins (17–19). Like ubiquitin, Urm1 is highly conserved in eukaryotes. While most Ubls are synthesized as an inactive precursor requiring protease cleavage before conjugation to substrates, Urm1 is directly produced as a mature protein without the need for protease cleavage (17). Unlike ubiquitination, which proceeds via a well-characterized three-step enzymatic process (the E1-E2-E3 cascade) (3, 4), urmylation appears to be less complex, requiring only the E1-like enzyme to generate covalently modified substrates (17). It is unclear whether the urmylation process is reversible since no proteolytic activity capable of reversing Urm1 conjugation has so far been identified (20, 21). In contrast to ubiquitination, urmylation does not appear to be a polymeric modification since poly-urmylation has not been observed in any eukaryotic cell (21). Urm1 is a dual-function protein that regulates the modification of tRNA thiolation as a sulfur carrier in addition to forming protein conjugates (22, 23). As compared to ubiquitin, Urm1 has fewer protein substrates, with only a few to a few dozens of substrate proteins identified in different eukaryotic organisms (24–26). The most well-known substrate is thioredoxin peroxidase Ahp1 (27). Both urmylated peroxiredoxin Ahp1 and tRNA thiolation contribute to the function of Urm1 in oxidative stress response (20, 21). Urm1 is a non-essential gene in *Saccharomyces cerevisiae*, but deletion of the gene results in sensitivity to various stressors, including nutrient deprivation, elevated temperature, and oxidant treatment (17, 27–29).

Ubls were originally thought to be confined to the eukaryotic domain of life. In 2010, the first archaeal *bona fide* ubiquitin-like modifications were identified in *Haloferax volcanii* (30). Three ubiquitin-like proteins, i.e., SAMP1, SAMP2, and SAMP3, in *H. volcanii* are conjugated to target proteins via isopeptide bonds, requiring only the E1-like UbaA enzyme, and their modification is reversed by JAB1/MPN/MOV34 metalloenzymes (30–35). Bioinformatic and structural studies have revealed that SAMPs are homologs of the eukaryotic Urm1 (36, 37). Furthermore, bioinformatic studies have shown that SAMPs are widely distributed across all archaeal phyla (36). In *H. volcanii*, 14, 28, and 23 conjugation sites were identified for SAMP2, SAMP3, and SAMP1, respectively, following *in vivo* exogenous overexpression of SAMPs (30, 35, 38). Many of the identified target proteins were associated with sulfur metabolism and oxidative stress. Among them, E1-like UbaA, SAMP2, and SAMP3 were sampylated, suggesting polySAMP chains could be formed in the cell. The *ubaA*, *samp1*, *samp2*, and *samp3* were not essential in *H. volcanii*. Growth of mutant strains, including the *ubaA* knockout as well as the single or triple knockouts of the Ubl genes (*samp1*, *samp2*, and *samp3*), was similar to that of the wild-type strain under standard culture conditions (32).

An Urm1/SAMP homolog has also been characterized in *Sulfolobus acidocaldarius* (39). The crystal structure of an Urm1 homolog from the related species *Saccharolobus solfataricus* reveals similarities to both eukaryotic Urm1 proteins and other archaeal SAMPs (18, 31, 37, 39, 40). In total, 29 distinct substrates were identified by using an *in vitro* approach, and 25 substrates were observed following the *in vivo* overproduction of Urm1. Modifications were also observed on the Urm1 protein itself, suggesting that polymeric chains could be formed. The urmylated substrates, including the modifier itself, were recognized and processed by the archaeal proteasome *in vitro* (39).

In the present study, we developed a highly efficient method for the proteome-wide analysis of sites of modification by the sole Ubl homolog Urm1 in *Saccharolobus islandicus*. This method entails the construction of a mutant *S. islandicus* strain encoding Urm1 with an H81R substitution, treatment of the cells with the proteasome inhibitor bortezomib, and affinity enrichment of urmylated peptides using an anti-K-ε-Gly-Gly antibody following peptide fractionation. By using this method, we were able to obtain the largest archaeal data set of *in vivo* Ubl substrates reported so far, which includes 783 conjugation sites from 330 proteins in *S. islandicus*. All seven lysine residues, except for K3, in Urm1 were modified, indicating the presence of poly-Urm1 chains in the cell. The modified proteins are involved in a range of biological processes, such as cell division, chromosomal organization, DNA replication, translation, proteasomal protein degradation, and sulfur relay. Urm1 was essential for the viability of *S. islandicus*, and the knockdown of *urm1* resulted in significant growth delay, accompanied by a drastic reduction in cellular concentration of cell division proteins (CdvB, CdvB1, CdvB2). Our data offer a novel view of the landscape and dynamics of Urm1-mediated protein modification and provide clues to the potential roles of protein urmylation in Archaea.

## MATERIALS AND METHODS

### Experimental design and statistical rationale

Three independent analyses of Urm1 conjugation sites in the proteome of *S. islandicus urm1* H81R strain were conducted. The data were analyzed using MaxQuant (v.1.6.15.0), and modified peptides were identified using a stringent standard, with detailed parameter settings provided in the subsequent sections on liquid chromatography tandem mass spectrometry (LC-MS/MS) analysis and data analysis. Detailed comparisons of the results from the three experiments (Exps.) are presented in the Results section. Due to the technical difficulties of the experiments, limited amounts of data were obtained from each experiment. The largest data set derived from the three experiments was therefore used to maximize the identification of modification sites. For KEGG pathway enrichment analysis, a Fisher’s exact test with a *P*-value <0.05 was performed. Statistical analysis of cell diameters from the control and *urm1* knockdown strains was conducted using a two-tailed unpaired *t*-test.

### Growth of organisms

*Saccharolobus* strains were generally grown in SCVy medium (41) at 75°C with shaking at 150 rpm. *S. islandicus* E233S (Δ*pyrEF*, Δ*lacS*) (42), a generous gift from Professor Qunxin She at Shandong University, was grown in SCVy supplemented with uracil (20 µg/mL). The *urm1*-silenced strain was grown in ACVy medium (43) to induce the silencing of *urm1*. In ACVy medium, arabinose replaced sucrose in SCVy medium, which facilitated plasmid transcription and subsequently induced silencing of *urm1*.

### Construction of the urm1 H81R strain

Genome-editing plasmid for the introduction of a point mutation (H81R) into *urm1* (SiRe_1713) was constructed by cloning a spacer with a sequence (5′-TAAATCATGGTGGTTAGAGATATCGAAGGTTCAAGTAAGT-3′) derived from the *urm1* gene and adjacent to the H81 codon, and a donor DNA sequence flanking the *urm1* gene into pSeRp as previously described (44). The donor fragment was then mutated at the PAM site and H81 codon successively with respective mutation primer pairs (Table S1), producing plasmid pGE-*urm1*. Plasmid pGE-*urm1* was introduced into *S. islandicus* E233S by electroporation (41). Transformed cells grown on SCVy plates were screened first by PCR amplification (Table S1). The resulting PCR products were subsequently analyzed by agarose gel electrophoresis and DNA sequencing. The desired mutant strain, denoted *urm1* H81R, was purified by repeated streaking.

### Construction of a *urm1* knockdown strain and a control strain

A protospacer with a protospacer adjacent sequence (PAS) motif at the 3′ end was chosen from the *urm1* gene sequence (Table S1). The spacer fragment was inserted into pSeRp at the BspMI site, producing pAC-*urm1-kd*. A *urm1*-silencing strain was obtained by transforming *S. islandicus* E233S with pAC-*urm1-kd* by electroporation (41). A control strain was constructed by transforming *S. islandicus* E233S with pSeRp. Transformed cells grown on SCVy plates were screened, and the target strains were purified by repeated streaking.

### Preparation of samples for the determination of the Urm1 conjugate proteome

The mutant strain *urm1* H81R was grown in SCVy medium containing uracil (20 µg/mL) at 75°C with shaking at 150 rpm. Cells were harvested at an optical density at 600 nm (OD_600_) of 0.3, resuspended in lysis buffer (8 M urea, 1% protease inhibitor mixture [Roche, Basel, Switzerland], 3 M trichostatin A, 50 mM nicotinamide, and 2 mM EDTA), and sonicated on ice. For Experiment 3, cells at an OD_600_ of 0.3 were treated with 10 µM bortezomib for 8 h, resuspended in lysis buffer, and sonicated on ice. After centrifugation at 20,000 × *g* for 30 min at 4°C, proteins in the supernatant were precipitated with ice-cold 20% trichloroacetic acid(TCA) for 2 h at 4°C. Following centrifugation at 15,000 × *g* for 10 min at 4°C, the pellet was washed three times with cold acetone. The proteins were dissolved in 8 M urea and 50 mM NH_4_HCO_3_, and the protein concentration was determined by using the bicinchoninic acid (BCA) kit (Beyotime, Shanghai, China). The protein solution was reduced with 5 mM dithiothreitol (DTT) for 30 min at 56°C and subsequently alkylated with 11 mM iodoacetamide for 15 min at 23°C in the dark. After the addition of 50 mM NH_4_HCO_3_ to lower the urea concentration to 2 M, trypsin was added at a trypsin-to-protein mass ratio of 1:50 and incubated overnight.

A sample of each resulting digest (2 and 4 mg of proteins for Experiments 1 and 2, respectively) was desalted, vacuum dried, and resuspended in immunoprecipitation (IP) buffer (50 mM Tris-HCl, pH 8.0, 1 mM EDTA, 100 mM NaCl, and 0.5% NP-40). For Experiment 3, tryptic peptides (16 mg of proteins) were fractionated into 72 fractions by high-pH reverse-phase high-performance liquid chromatography(HPLC) on a C18 column (BetaSil C18, 5 µm particles, 10 mm ID × 250 mm, Thermo Fisher). These fractions were combined into four pools, vacuum dried, and dissolved in IP buffer. Peptides from each pool were subjected to peptide enrichment separately and incubated with pre-washed anti-K-ε-Gly-Gly antibody beads (PTM-1104, PM Biolabs, Hangzhou, China) at 4°C for overnight with gentle shaking. To remove nonspecifically bound peptides, the beads were washed four times with 1 mL of IP buffer and twice with deionized H_2_O. The peptides were eluted by washing three times with 0.1% trifluoroacetic acid. The eluted fractions were combined, vacuum-dried, and desalted by C18 ZipTips.

### LC-MS/MS analysis and data analysis

LC-MS/MS analysis was performed on a nanoElute ultra performance liquid chromatography(UPLC) system (Bruker Daltonics, Germany) coupled online to a timsTOF Pro mass spectrometer (Bruker Daltonics, Germany). Peptides were loaded onto an in-house-manufactured 15 cm fritless column packed with C18 resin (1.9 µm particles, Dr. Maisch GmbH, Germany) and eluted at a flow rate of 450 nL/min with a gradient of solvent A (0.1% formic acid and 2% acetonitrile in water) to solvent B (0.1% formic acid in acetonitrile): 6% to 42% B over 42 min, 24% to 32% B over 14 min, and 32% to 80% B over 2 min. The eluted peptides were subjected to analysis on a timsTOF Pro mass spectrometer. MS acquisition was operated in data-dependent parallel accumulation-serial fragmentation (PASEF) mode with 10 PASEF MS/MS frames in one complete frame. The capillary voltage was set to 1.6 kV, and the MS and MS/MS spectra were acquired from 100 to 1,700 m/z. Dynamic exclusion was set to 30 s.

The RAW mass spectrometry files were processed using MaxQuant (v.1.6.15.0) with an integrated Andromeda search engine. Tandem mass spectra were searched against the Uniprot *S. islandicus* REY15A database (2018/12/26, taxonomy ID: 930945, 2,631 sequences) concatenated with a reverse decoy database. Mass tolerance for precursor ions was set at 20 ppm for first search and main search, and the mass tolerance for fragment ions was set at 20 ppm. Trypsin/P was specified as the cleavage enzyme, allowing up to four missing cleavages. The minimum peptide length was set at 7. Interference filters were employed by setting peptide isolation factor(PIF) at 0.75. Carbamidomethyl (C) was set as a fixed modification, whereas acetylation (N-terminus), oxidation (M), and GlyGly (K) were set as variable modifications. False discovery rate was adjusted to 1%. Modified peptides were identified with cutoffs set at *P*-value <0.05 and score >40. Localization probability was set at >0.75.

### Bioinformatic analysis

All identified sequences of Urm1 conjugation sites were analyzed by MoMo software (https://meme-suite.org/meme/tools/momo). arCOG annotation was performed based on the arCOG database (https://ftp.ncbi.nih.gov/pub/wolf/COGs/arCOG/). KEGG annotation was conducted using BlastKOALA (www.kegg.jp/blastkoala/), and significantly enriched pathways were identified with cutoffs set at *P*-value (Fisher’s exact test) <0.05. Protein-protein interaction analysis was performed using STRING (https://cn.string-db.org/cgi/input).

### Immunoblotting

Cells were subjected to electrophoresis in an 8%–18% gradient SDS-PAGE gel. Proteins in the gel were transferred onto a polyvinylidene fluoride (PVDF) membrane (Merck Millipore). The membrane was incubated first with an indicated rabbit antibody and then with the anti-rabbit antibody conjugated to horseradish peroxidase(HRP) (Promega). Following the addition of the enhanced chemiluminescence Western blot substrate (Thermo Fisher Scientific), target proteins were visualized with a Tanon 5200 Multi Chemiluminescent System (Tanon, Shanghai, China). The antibody against Urm1 was prepared in this study, as detailed in Fig. S1. The antibodies against CdvB, CdvB1, and CdvB2 were generously provided by Professors Qunxin She and Yulong Shen at Shandong University.

### Transmission electron microscopy (TEM)

Cells were stained with 2% (wt/vol) uranyl acetate and observed under a JEM-1400 (JEOL) TEM (45). The diameter of a cell was measured using ImageJ (v.1.54 g).

## RESULTS

### Proteome-wide identification of Urm1 conjugation sites

**Fig 1:**
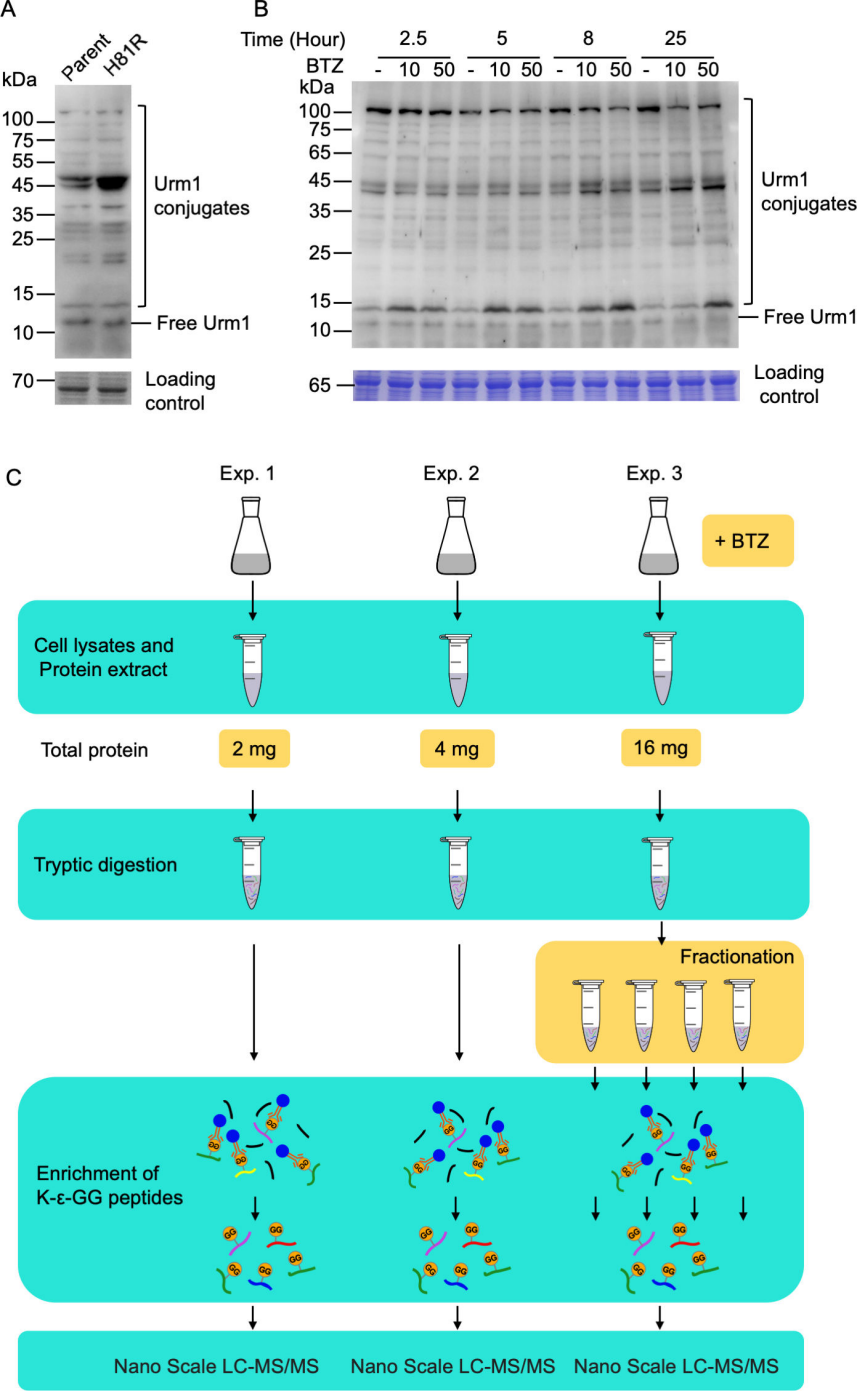
Detection of Urm1 conjugates by immunoblotting and proteomics. (**A**) Detection of Urm1 conjugates in the *urm1* H81R mutant strain and the parent strain. Equal amounts of the cell lysates of the parent strain (wild-type *urm1*) and *urm1* H81R strain were resolved by SDS-PAGE and subjected to immunoblotting using anti-Urm1 antibody prepared as described in Fig. S2. Parallel samples at the same amounts as those above were subjected to SDS-PAGE and stained with Coomassie brilliant blue G250 to use as a loading control (lower panel). Urm1 conjugates and free Urm1 are indicated. (**B**) Effect of the proteasome inhibitor bortezomib on Urm1 conjugation in H81R. H81R cells were grown to the exponential phase. Bortezomib (BTZ, 10 or 50 µM) was added, and the cells were collected after incubation for 2.5, 5, 8, and 25 h. Equal amounts of the cell lysates were subjected to immunoblotting using anti-Urm1 antibody. Urm1 conjugates and free Urm1 are shown. Parallel samples, processed as above, were used to produce a loading control (lower panel). (**C**) Workflow of the detection of a large-scale Urm1 conjugation proteome. The *urm1* H81R strain was incubated under the same growth conditions in all three experiments except for the addition of bortezomib in Exp. 3. Total proteins for Exps. 1, 2, and 3 were 2, 4, and 16 mg, respectively. The proteins were digested with trypsin, and the resulting peptides were affinity enriched by using the anti-K-ε-Gly-Gly antibody. In Exp. 3, peptides were fractioned before affinity enrichment. The enriched peptides were pooled and analyzed by MS.

We started by establishing a procedure for the identification of Urm1 conjugation sites. The procedure involves the enrichment of urmylated peptides with an anti-K-ε-Gly-Gly-specific antibody. Unlike ubiquitin, which typically contains a Lys or Arg residue before the two C-terminal Gly residues, allowing the exposure of a di-glycyl remnant at the modified site after trypsin digestion, nearly all Urm1 homologs, including that from *S. islandicus*, encode a His before the two C-terminal Gly residues. Although the peptide bond at the carboxyl end of the His residue can be cleaved by chymotrypsin (39), the cleavage efficiency is extremely low, making chymotrypsin digestion unsuitable for a proteomic approach designed to identify Urm1 conjugation sites. Since far fewer PTMs have been described for Arg than for Lys (46), we mutated H81, the His before the two C-terminal Gly residues in the *S. islandicus* Urm1, into Arg through site-directed mutagenesis to permit efficient cleavage by trypsin. The H81R mutation did not affect cell growth or result in significant changes in the profile of the Urm1 conjugates of *S. islandicus* (Fig. S1; Fig. 1A). Therefore, we conducted all of our three large-scale proteomics experiments (Exps. 1–3) in the H81R mutant strain (Fig. 1C).

**Fig 2:**
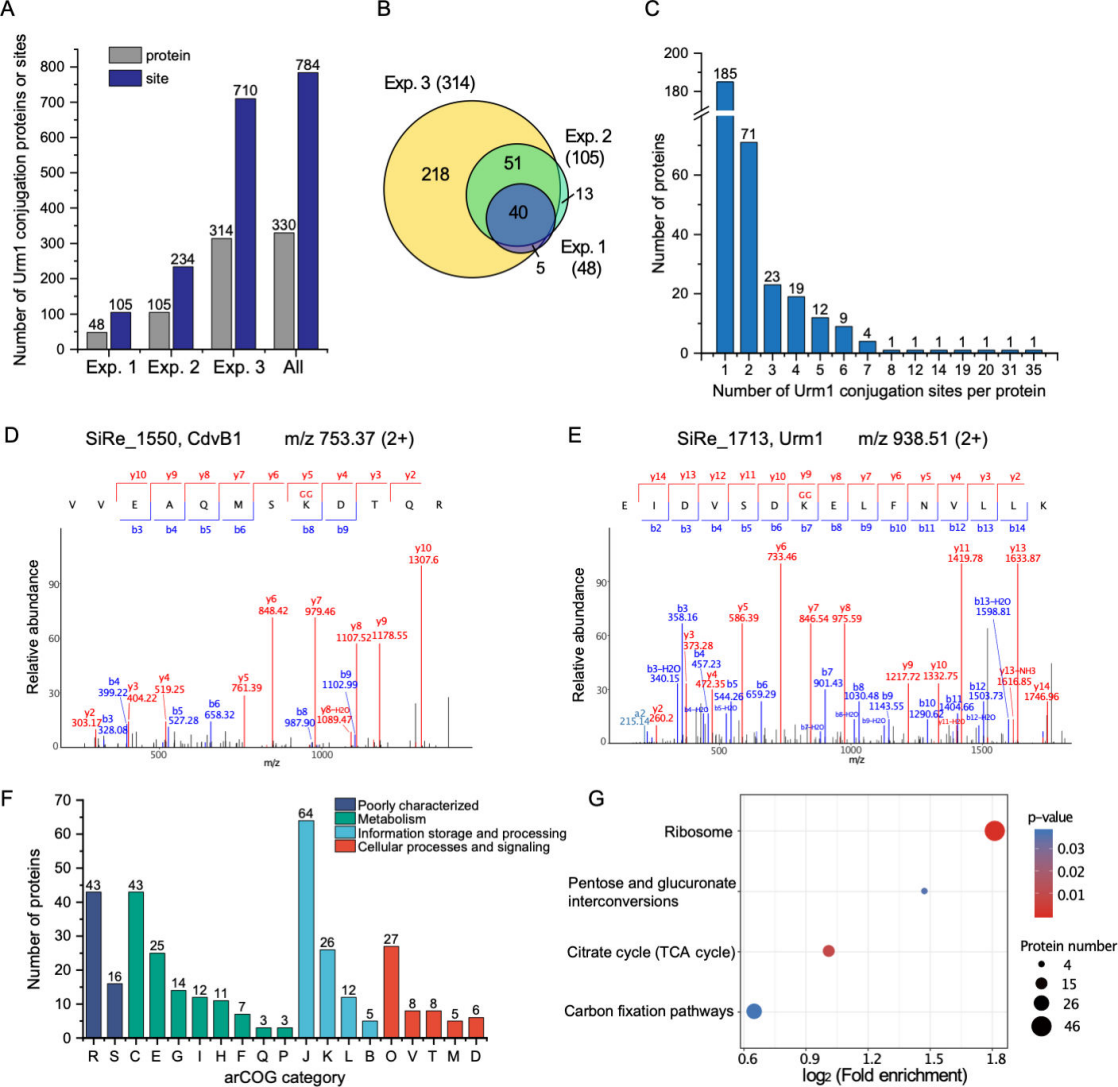
Characterization of Urm1 targets. (**A**) Urm1 conjugation sites and proteins identified in the three experiments. (**B**) A Venn diagram showing the number and relationship of Urm1 conjugation proteins identified in the three experiments. (**C**) Number of Urm1 conjugation sites per protein. (**D, E**) MS^2^ spectra of ^107^VVEAQMSK^GG^DTQR^118^ from CdvB1 (SiRe_1550) (**D**) and ^18^EIDVSDK^GG^ELFNVLLK^32^ from Urm1 (SiRe_1713) (**E**). The m/z values and charge of the precursor ions are indicated in each panel (top right). Spectra show the annotated peaks from the C-terminal y (red) and N-terminal b (blue) fragment ions. The m/z values of the precursor ions and the values of the fragment ions reveal that CdvB1 K114 and Urm1 K24 were modified by di-glycine. Dehydrated ions and deaminized ions are indicated. GG, di-glycine. (**F**) arCOG analysis of Urm1 target proteins. R, general function prediction only; S, function unknown; C, energy production and conversion; E, amino acid transport and metabolism; G, carbohydrate transport and metabolism; I, lipid transport and metabolism; H, coenzyme transport and metabolism; F, nucleotide transport and metabolism; Q, secondary metabolites biosynthesis, transport, and catabolism; P, inorganic ion transport and metabolism; J, translation, ribosomal structure, and biogenesis; K, transcription; L, replication, recombination, and repair; B, chromatin structure and dynamics; O, post-translational modification, protein turnover, and chaperones; V, defense mechanisms; T, signal transduction mechanisms; M, cell wall/membrane/envelope biogenesis; D, cell cycle control, cell division, and chromosome partitioning. (**G**) KEGG analysis of the Urm1 target proteins. Significantly enriched pathways were identified with cutoffs set at *P*-value <0.05 (Fisher’s exact test). The fold enrichment refers to the proportion of identified modified proteins within this functional category compared to the proportion of all proteins in the species database.

We also determined the effect of the proteasome inhibitor bortezomib on the level of protein urmylation in the cell. We found that the intensity of Urm1 conjugates gradually increased with the exposure of the cells to the proteasome inhibitor bortezomib over a period from 2.5 to 25 h (Fig. 1B). Treatment with bortezomib at either 10 or 50 µM enhanced the overall intensity of Urm1 conjugates without producing new conjugates. In addition, we found that more Urm1 conjugates were obtained when increasing amounts of proteins were assayed (Fig. 2A and B; Table S2). These findings prompted changes in our protocol for Exp. 3 to include the pretreatment of the cells with bortezomib, use of more cellular proteins, and pre-enrichment separation of peptides (Fig. 1C).

As shown in Fig. 2A, Exp. 3 revealed more modification sites than the other two experiments (Exps. 1 and 2). Moreover, the sites found in Exps. 1 and 2 were almost all covered in Exp. 3 (Fig. 2B). While these results indicate the reliability and reproducibility of the assays, they also suggest that the number of the identified sites of Urm1 conjugation likely remains an underestimate as it increased with the amount of the sample protein analyzed. The di-glycine motif from the Urm1 is attached via an isopeptide bond to the ε-amino group of the lysine residue at the modification site, as exemplified by two representative MS^2^ spectra, i.e., VVEAQMSK^GG^DTQR (Fig. 2D) and EIDVSDK^GG^ELFNVLLK (Fig. 2E), which are mapped to CdvB1 (SiRe_1550) and Urm1 (SiRe_1713), respectively. A total of 783 unique sites of Urm1 conjugation mapped to 330 proteins, including 679 sites with unambiguous positions and 104 sites with ambiguous positions, were identified in the three experiments (Fig. 2A; Table S2). In comparison, only 62 Urm1 sites in 47 proteins were identified by a combination of *in vitro* and *in vivo* approaches in *S. acidocaldarius* (39), which is phylogenetically close to *S. islandicus*. It is noticed that 778 of these sites, corresponding to 311 proteins, in *S. islandicus* were not reported previously.

Approximately 97% (319/330) of the urmylated proteins contain no more than six conjugation sites, with proteins harboring a single site being the most abundant (56%, 185/330) (Fig. 2C). Six proteins, i.e., glycine–tRNA ligase (SiRe_1559), peroxiredoxin family/AhpE-like subfamily (SiRe_2592), glutamate dehydrogenase (SiRe_0689), elongation factor 1-alpha (SiRe_1776), thermosome (SiRe_1214), and thermosome (SiRe_1716), were urmylated at more than 10 sites (Table S2). The two urmylated thermosome proteins were modified most extensively, with the numbers of modification sites reaching 31 (SiRe_1214) and 35 (SiRe_1716), respectively. It is worth noting that simultaneous urmylation at two or three sites in a single peptide was detected (Table S2). To determine the sequence preference of lysine urmylation, we examined the sequence spanning from position −10 to +10 in relation to each urmylated lysine. A conserved Ala or Thr at position −2 or a conserved Glu at position −3 was found in 15.5% or 11.5%, respectively, of all identified urmylated peptides (Fig. S3).

### Characterization of Urm1 target proteins

To gain insights into the role of protein urmylation in the physiology of *S. islandicus*, we examined the identified substrates using arCOG category, KEGG pathway, and STRING interaction network analyses. As revealed by arCOG category analysis, the Urm1 substrates were significantly enriched in translation, ribosomal structure, and biogenesis (arCOG J) (Fig. 2F). A total of 46 ribosomal proteins, which accounted for 72% of the total ribosomal proteins encoded by the genome, were urmylated. Modified proteins were also enriched in cellular metabolism, especially in energy production and conversion (arCOG C), amino acid transport and metabolism (arCOG E), transcription (arCOG K), post-translational modification, protein turnover, and chaperones (arCOG O). Eight chaperones, including three Hsp60 family thermosome proteins (SiRe_1214, SiRe_1716, and SiRe_2245), small heat shock protein Hsp20 (SiRe_0216), prefoldin β-subunit (SiRe_1279), thioredoxin (SiRe_1631), and two peroxiredoxin family proteins (SiRe_2592 and SiRe_0067), were among the preferred modification targets. Both peroxiredoxin family proteins were homologs of yeast peroxiredoxin Ahp1, suggesting that *S. islandicus* Urm1 may play a role in oxidative stress response. Several proteins from the sulfur relay system, including the MoaE-MoaD fusion protein (SiRe_0186) in Moco biosynthesis, sulfurtransferase ThiI (SiRe_1665) in thiamine biosynthesis, and sulfurtransferase TusA (SiRe_0659) in tRNA 2-thiouridine biosynthesis, were also urmylated.

**Fig 3:**
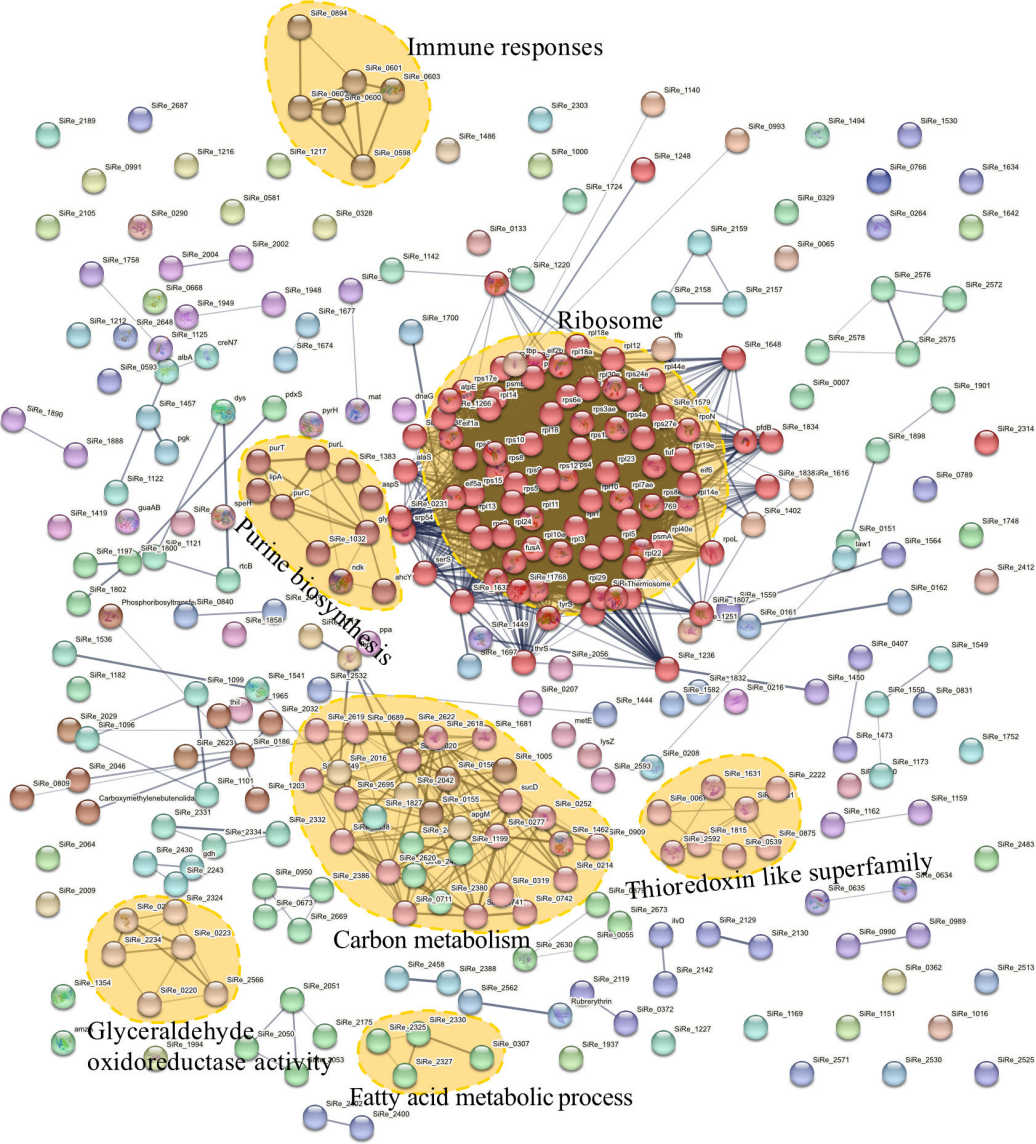
Protein-protein interaction analysis of Urm1 targets by STRING. Networks of Urm1 targets associated with ribosome, carbon metabolism, immune response, purine biosynthesis, thioredoxin-like superfamily, glyceraldehyde oxidoreductase activity, and fatty acid metabolism are indicated.

KEGG pathway analysis identified four significantly enriched pathways, i.e., ribosome, pentose and glucuronate interconversions, the TCA cycle, and the carbon fixation pathway (Fig. 2G). Apart from the ribosome, the remaining three pathways were all involved in energy metabolism. Protein-protein interaction analysis using the STRING database clustered Urm1 conjugation targets into several distinctive functional networks (Fig. 3). In agreement with the results of the arCOG and KEGG analyses, ribosome represents the biggest cluster. Moreover, a number of Urm1 substrates were associated with the network of carbon metabolism, which includes proteins of the TCA cycle, carbon fixation pathway, and pyruvate metabolism. Urm1 substrates were also found in networks associated with immune responses, purine biosynthesis, fatty acid metabolic processes, thioredoxin-like superfamily, and glyceraldehyde oxidoreductase activity.

Of particular interest is the observation that cell division proteins CdvA (SiRe_1173), CdvB1 (SiRe_1550), and CdvB2 (SiRe_1200) were urmylated at one, three, and four sites, respectively (Table 1). The proteins involved in the Urm1-conjugation pathway, such as Urm1 (SiRe_1713), E1-like enzyme Uba4p (SiRe_1697), as well as proteasome α-subunit (SiRe_1271) and β-subunit (SiRe_1237), were also modified. Small nucleic acid-binding protein Cren7 (SiRe_1111), Sis10b (SiRe_1125), Sis10b2 (SiRe_1123), Sul7d (SiRe_0668, SiRe_2648), and SSB (SiRe_0161) were modified at two to eight lysine residues. The modified proteins are associated with various fundamental biological processes, including cell division, chromosomal organization, DNA replication, translation, proteasomal protein degradation, and sulfur relay.

**TABLE 1:** Selected Urm1 target proteins involved in fundamental biological processes

Protein	Locus tag	Position (K)	Function
Cell division protein			
CdvA	SiRe_1173	260	Cell division
CdvB1	SiRe_1550	53, 114, 221	Cell division
CdvB2	SiRe_1200	11, 70, 78, 185	Cell division
Urm1-proteasome pathway			
Urm1	SiRe_1713	7, 24, 32, 37, 48, 58	Urm1-conjugation pathway
Uba4p	SiRe_1697	305	Urm1-conjugation pathway
Proteasome β- subunit	SiRe_1237	173	Proteasome
Proteasome α-subunit	SiRe_1271	34^*a*^, 35^*a*^, 43^*a*^, 45, 54, 56, 204	Proteasome
Nucleic acid-binding protein			
Cren7	SiRe_1111	16, 24, 31, 42, 48, 53	Chromatin protein
Sis10b	SiRe_1125	16^*a*^, 17^*a*^, 48, 64, 68	DNA/RNA-binding protein
Sis10b2	SiRe_1123	4, 45	DNA/RNA-binding protein
Sul7d^*b*^	SiRe_0668, SiRe_2648	13, 19, 28, 40, 49, 53, 61, 63^*a*^	DNA-binding protein
SSB	SiRe_0161	64, 70, 90, 92^*a*^	DNA replication, recombination, and repair

^*a*^ Indicated peptides are modified, but the locations of the marked modification sites remain uncertain. Detailed information for each site is provided in Table S2.

^*b*^ It is unclear which of the two copies of Sul7d (SiRe_2648 and SiRe_0668) gave rise to the identified urmylated peptides because of the high similarity in sequence between the two proteins in *S. islandicus*.

### Poly-urmylation

**Fig 4:**
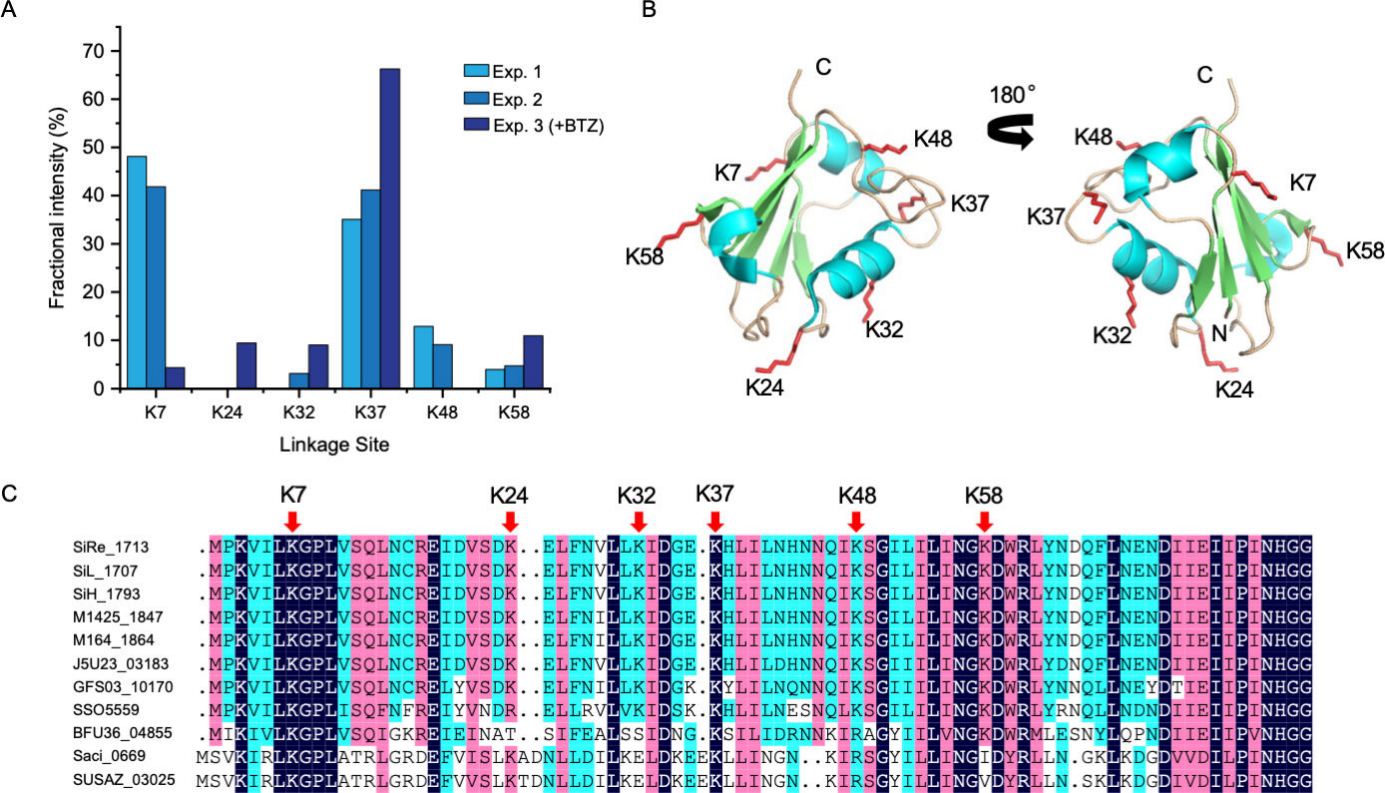
Analysis of poly-urmylation. (**A**) Occurrence of linkage at different sites of Urm1. The fraction of linkage at a specific site in total linkage on Urm1 is shown. (**B**) The 3D representation of the positions of linkage sites of Urm1. The 3D structure of *S. islandicus* Urm1 was derived from the known structure of *S. solfataricus* Urm1 (PDB ID: 4WWM). The two closely related Urm1 homologs share 78% similarity at the amino acid sequence level and possess six lysine residues at the same positions with the exception of Arg, instead of Lys, at position 24 in *S. solfataricus* Urm1. α helices, β strands, and loops are shown in cyan, green, and tan, respectively. The side chains of the modified lysine are shown in red. N, N terminus; C, C terminus. (**C**) Sequence alignment of Urm1 homologs from *Saccharolobus* and *Sulfolobus* species. Linkage sites identified in *S. islandicus* are indicated with red arrows. Sequences are from *S. islandicus* REY15A (SiRe_1713), *S. islandicus* LAL14/1 (SiL_1707), *S. islandicus* HVE 10/4 (SiH_1793), *S. islandicus* M.14.25 (M1425_1847), *S. islandicus* M.16.4 (M164_1864), *S. shibatae* (J5U23_03183), *S.* sp. E5-1-F (GFS03_10170), *S. solfataricus* P2 (SSO5559), *S.* sp. A20 (BFU36_04855), *S. acidocaldarius* DSM 639 (Saci_0669), and *S. acidocaldarius* SUSAZ (SUSAZ_03025).

Numerous Urm1 peptides were found to be modified by a di-glycine motif (Table 1; Table S2), indicating the presence of abundant Urm1 linkages. Linkages to six of the seven Urm1 lysine residues were detected, with a representative MS^2^ spectrum for each site shown in Fig. 2E and Fig. S4. To assess the occupancy of each linkage site, we analyzed its fractional intensity, a measure of the contribution of linkage at a specific site to the overall linkages on Urm1 (Fig. 4A; Table S3). Modification shows a strong preference for K7 and K37, a moderate preference for K48, and a low preference for K58 and K32 under our growth conditions. Intriguingly, the K37 linkage became overwhelmingly predominant in the presence of bortezomib, suggesting that this linkage triggered protein degradation via proteasome. Additionally, the K24 linkage appeared exclusively following bortezomib treatment. Structural mapping showed that all of the six Urm1 linkage sites were exposed on the surface of Urm1 (Fig. 4B), while K3, the only unmodified lysine residue, was buried within the protein (Fig. S5). The steric hindrance may explain the lack of modification at K3. Sequence alignment shows that the most preferentially modified sites, K7 and K37, are highly conserved, and the other sites are moderately conserved among *Saccharolobus* and *Sulfolobus* Urm1 proteins (Fig. 4C). Therefore, the extent of modification at a site depends on its sequence conservation and structural accessibility.

### Response of urmylation to growth conditions and stress treatments

**Fig 5:**
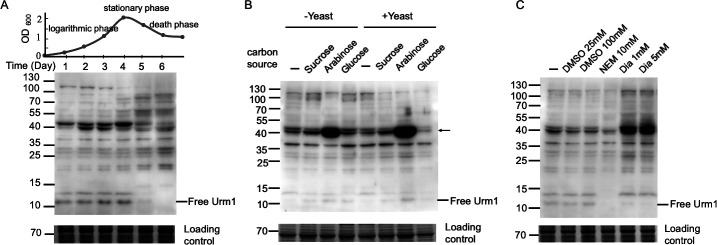
Protein urmylation in *S. islandicus* under various growth conditions or in response to different oxidant treatments. (**A**) Protein urmylation in *S. islandicus* growing in different growth phases. *S. islandicus* E233S was grown in SCVy medium, and samples were taken at time points. The growth curve is shown (top panel). Cells were harvested and resuspended to the same OD_600_. An equal aliquot of each sample was subjected to immunoblotting using anti-Urm1 antibody (middle panel). Parallel samples were subjected to SDS-PAGE and stained with Coomassie brilliant blue G250 for use as a loading control (bottom panel). (**B**) Effect of carbon sources on protein urmylation in *S. islandicus. S. islandicus* E233S was grown in the mineral salts (41) supplemented with casein and vitamins as well as yeast extracts and carbohydrate, as indicated. The exponentially growing cells were harvested and subjected to immunoblotting using anti-Urm1 antibody, as described above. The most intensely stained band is indicated. The loading control was prepared as described above. (**C**) Effect of oxidants on protein urmylation in *S. islandicus*. Exponentially growing *S. islandicus* E233S cells were treated with 25 mM dimethyl sulfoxide (DMSO), 100 mM DMSO, 10 mM N-ethylmaleimide (NEM), 1 mM, or 5 mM diamide for 2 h. Cells were harvested and subjected to immunoblotting using anti-Urm1 antibody, as described above. The loading control was prepared as described above.

Ubls modification in *H. volcanii* and yeast is affected by growth conditions and stress treatments (25, 28, 30, 47, 48). To determine the growth-dependent profile of protein urmylation, we collected *S. islandicus* cells growing in SCVy medium at various growth phases and detected Urm1 conjugates in the cells by immunoblotting using anti-Urm1 antibody (Fig. 5A). The pattern of protein urmylation in the death phase was significantly different from those in the logarithmic and stationary phases (Fig. 5A). While some proteins remained similarly urmylated throughout the growth cycle, others were more growth phase dependent. Free Urm1 was abundant in the logarithmic and stationary phases but disappeared in the death phase, suggesting a possible link between the presence of free Urm1 and cellular activity.

We also determined the effect of different growth conditions and treatments on protein urmylation in the cells. The cells were grown to the logarithmic phase in minimal medium with glucose, arabinose, or sucrose used as the carbon source. The general patterns of protein urmylation in these cells were similar except that an unidentified ~40 kDa protein was more heavily urmylated in the presence of arabinose than in the presence of either glucose or sucrose (Fig. 5B). We then subjected the exponentially growing cells to treatment with various oxidants, including the mild oxidant dimethyl sulfoxide, the strong oxidant H_2_O_2_, paraquat, N-ethylmaleimide, and diamide (Fig. 5C; Fig. S6). Diamide was the sole agent which significantly increased the overall intensity of Urm1 conjugates while simultaneously reducing the level of free Urm1 in the cells (Fig. 5C; Fig. S7). Since diamide is known to specifically oxidize thiols to disulfide bonds in proteins, our results suggest that protein urmylation is sensitive to thiol oxidation.

### Characterization of a *urm1* knockdown strain of *S. islandicus*

To look into the physiological function of urmylation, we attempted to create a *urm1* or a *uba4p* deletion mutant. However, these efforts were unsuccessful, suggesting that the two genes were essential for the viability of the organism. This agrees with the results of the essentiality assays conducted in the closely related strain *S. islandicus* M.16.4 by Zhang et al. (49). Therefore, we constructed an *urm1* knockdown strain, referred to as *urm1-kd*, by cloning a spacer sequence targeting *urm1* in a gene editing plasmid under the control of the arabinose promoter and transforming *S. islandicus* with the plasmid.

**Fig 6:**
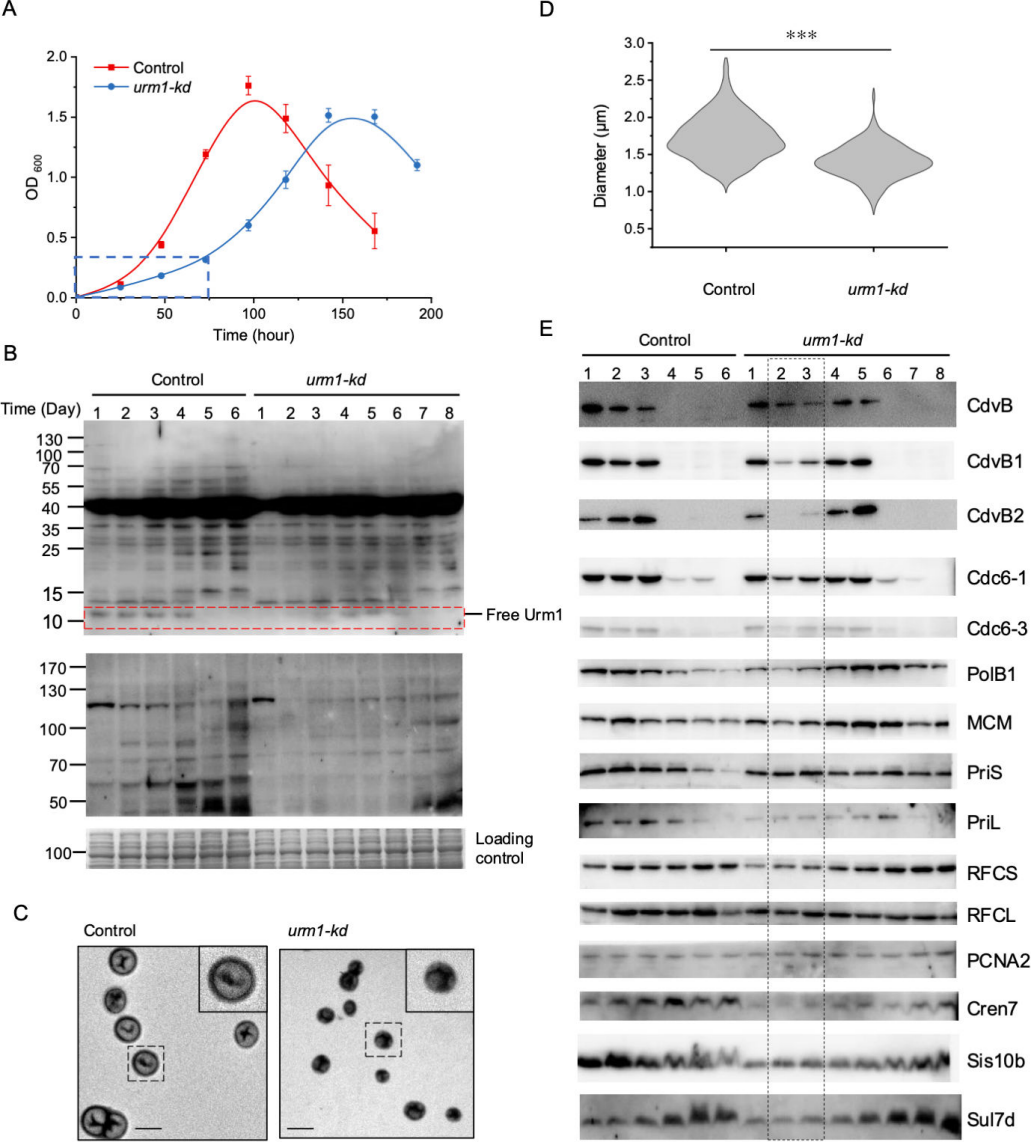
Characterization of the *urm1-kd* strain. (**A**) Growth curve of *urm1-kd*. The *urm1-kd* and the control strains were incubated in ACVy medium. All data points are an average of three independent measurements. The period of extremely low growth rate for *urm1-kd* was boxed in blue. (**B**) Protein urmylation in *urm1-kd*. Samples of the *urm1-kd* and the control strains, taken at time points during the growth, as shown in panel A, were adjusted to the same OD_600_ of 20, and the OD-adjusted samples were subjected to 8%–18% gradient SDS-PAGE (top panel) and 10% SDS-PAGE (middle panel). Urmylated proteins in the gradient gel and the >40 kDa portion of the 10% SDS-PAGE gel were detected by immunoblotting using anti-Urm1 antibody. Free Urm1 was boxed in red. A control for potential variation in the amount of cells among the OD-adjusted samples was prepared by resolving the same set of the samples as that used in the above immunoblotting assays on a 15% SDS-PAGE gel and staining the gel with Coomassie Brilliant Blue (bottom panel). (**C**) TEM analysis of the *urm1-kd*. Exponentially growing cells of the *urm1-kd* and the control strains were observed under TEM. Inset, an enlarged image of a cell. Scale, 2 µm. (**D**) Cell sizes of the *urm1-kd* and the control strains. Cells were observed under TEM, and the size of a cell was measured using ImageJ software (*n* > 200, three biological replicates). The mean of the cell diameter of each strain is indicated. ***, statistically significant differences (significance level: *P*-value <0.001, two-tailed unpaired *t*-test). (**E**) Cellular contents of selected proteins involved in cell division (CdvB, CdvB1, and CdvB2), DNA replication (Cdc6-1, Cdc6-3, PolB1, MCM, PriS, PriL, RFCS, RFCL, and PCNA2), and nucleic acid binding (Cren7, Sis10b, and Sul7d) in the *urm1-kd* and the control strains. The same set of samples as that in panel B was subjected to immunoblotting with each of the indicated antibodies.

We then examined the growth phenotype of the mutant strain. After growth in SCVy medium, *urm1-kd*, along with the control strain, was inoculated into ACVy medium in which arabinose replaced the sucrose in SCVy medium to induce the gene editing plasmid. The cellular level of Urm1 in the exponentially growing *urm1-kd* cells was about 23% of that of the control strain (Fig. S8). As shown in Fig. 6A, *urm1-kd* exhibited significant growth delay compared to the control strain and started to grow rapidly with a maximum growth rate only slightly lower than that of the control strain after incubation for 3 days (Fig. 6A). Protein urmylation in both the knockdown and the control strains was determined by immunoblotting using anti-Urm1 antibody. As shown in Fig. 6B, a heavily urmylated ~40 kDa protein appeared under ACVy medium, consistent with the results shown in Fig. 5B. Notably, the staining intensity of higher molecular weight (higher-MW) urmylation bands from *urm1-kd* was substantially lower than that from the control strain (Fig. 6B, upper panel). This observation is supported by an immunoblot with better resolved higher-MW bands (Fig. 6B, middle panel). By comparison, the staining intensity of lower-MW urmylation bands from the knockdown strain was less drastically lower than that from the control strain (Fig. 6B, upper panel). Notably, in the control strain, free Urm1 was maintained at a high and constant level from the beginning of the incubation until after the start of the death phase, during which the protein became hardly detectable, whereas urmylated proteins gradually increased throughout the entire growth cycle. On the other hand, free Urm1 in *urm1-kd* was undetectable during early incubation and began to accumulate significantly from day 4, coinciding with the start of rapid growth of the strain. Free Urm1 remained relatively abundant until the death phase. Therefore, it appears that the presence of free Urm1 is required for the growth of the organism.

Morphological analysis by TEM showed that the cell size of the *urm1-kd* strain during the growth delay period was smaller than that of the control strain (Fig. 6C). Cell size measurement reveals that the *urm1-kd* and control cells were 1.4 and 1.7 µm in size on average (*n* > 200, three biological replicates) during the exponential growth phase (Fig. 6D). The change in cell size may be attributed to defects in or the altered cellular levels of cell division proteins, as found in the *cdvB2* deletion mutant of *S. acidocaldarius* (50) and the *cdvABC* knockdown strain of *S. solfataricus* (51).

The availability of the *urm1-kd* strain permitted an analysis of the impact of the change in the intracellular level of Urm1 on the *S. islandicus* proteome. A label-free quantitative proteomic analysis was performed on both the *urm1-kd* and control strains. A total of 1,910 quantifiable proteins, accounting for 73% of the proteins encoded by the genome (2,631), were identified (Table S4). When a cutoff was set at a fold change of ≥1.50, there were 99 upregulated and 80 downregulated proteins, and they represented ~7% of the quantified proteins (Table S4; Fig. S9A). As revealed by arCOG analysis, the differentially regulated proteins in the *urm1-kd* strain belong to multiple functional categories, such as amino acid and ion transport, translation, ribosomal biogenesis, transcription, energy production, and defense (Fig. S9B). Therefore, Urm1 appears to play a regulatory role in diverse biological processes.

We then sought to further understand the effect of *urm1* knockdown on selected fundamental biological processes in *S. islandicus*. The cellular contents of proteins involved in cell division, DNA replication, and nucleic acid binding were assessed (Fig. 6E). In the control strain, the cell division proteins CdvB, CdvB1, and CdvB2, the DNA replication initiators Cdc6-1 and Cdc6-3, as well as the DNA replication proteins PolB1 and MCM were present in abundance in the exponential phase (days 1–3), peaking on day 3, and no longer existed or drastically diminished in the stationary and death phases (days 4–6). Free Urm1 was abundant and maintained at a constant level until the start of the death phase, during which the protein became undetectable. Strikingly, the levels of the cell division proteins, especially CdvB2 and, to a lesser extent, CdvB1, decreased drastically in *urm1-kd* during the period of 2 day growth delay (days 2 and 3). It is noticed that little free Urm1 remained immediately following the change of growth medium from SCVy to ACVy to turn down the transcription of *urm1*, and the protein slowly increased in concentration during the growth delay, eventually reaching the level slightly lower than that in the exponentially growing control strain. CdvB, CdvB1, and CdvB2, together with CdvA and CdvC, comprise the Cdv system in *Sulfolobales*, which drives cell division. Moderate decreases were also observed in the cellular contents of Cdc6-1, Cdc6-3, PolB1, and MCM in the knockdown mutant. These cell division and DNA replication proteins started to increase in cellular content only after the growth delay. On the other hand, no significant drops in cellular level were found for PriS, RFCS, RFCL, PCNA2, or Sul7d in the knockdown strain. The other tested proteins (i.e., PriL, Cren7, and Sis10b) were less abundant in the *urm1-kd* strain than in the control strain. Therefore, we speculate that the proteins involved in cell division and DNA replication (i.e., CdvB, CdvB1, CdvB2, Cdc6-1, Cdc6-3, PolB1, and MCM), particularly CdvB2, are under the control involving Urm1, and the downregulation of these proteins in *urm1-kd* presumably is part of the response of the cell to growth retardation resulting from the lack of the optimal amounts of free Urm1. It is noticed that the cellular contents of these proteins in *urm1-kd* were comparable to those in the control strain on day 1, presumably due to the lag in the impact of the cellular processes regulated by free Urm1 on the turnover of these proteins.

## DISCUSSION

In this study, we developed a proteomic method for the effective identification of *bona fide* Ubl substrates *in vivo* in *S. islandicus*. This method involves the construction of an *S. islandicus* mutant strain encoding Urm1 with an H81R substitution, treatment of the strain with the proteasome inhibitor bortezomib, and affinity enrichment of the urmylated peptides with an anti-K-ε-Gly-Gly antibody following peptide fractionation and. As many as 783 Urm1 conjugation sites, mapped to 330 proteins, were identified. These numbers are far greater than those reported in *H. volcanii* and *S. acidocaldarius,* presumably due to the increased sensitivity of the method (30, 35, 38, 39). Our results indicate that protein urmylation is widespread in *S. islandicus*, with the number of substrates comparable to those of protein methylation (52), acetylation (53), and phosphorylation (54, 55) in this archaeon. Therefore, our observation argues against the notion that Urm1 homologs modify only a limited number of substrates (20, 21), and will permit a better understanding of the functions of protein urmylation in Archaea.

We present solid evidence in support of the formation of diverse poly-urmylation chains by archaeal Urm1 *in vivo*. Of the seven lysine residues in *S. islandicus* Urm1, six (K7, K24, K32, K37, K48, K58) are sites of poly-urmylation, and the remaining lysine K3 is not found to be urmylated. K7 and K37 were the predominant linkage sites in cells growing under our experimental conditions, but K37 became the only major linkage site after treatment of the cells with the proteasome inhibitor bortezomib. Therefore, we speculate that poly-urmylation via K37 targets proteins for proteasomal degradation. *S. acidocaldarius* Urm1 was shown to be self-modified at K25, K38, and K70 *in vivo* and *in vitro* (39). As revealed by sequence alignment, K25 in *S. acidocaldarius* Urm1 corresponds to K24, a non-preferred linkage site, in *S. islandicus* Urm1, whereas no lysine corresponding to either K38 or K70 in the *S. acidocaldarius* Urm1 exists in the *S. islandicus* Urm1. Therefore, sites of modification in the Urm1 proteins from the two phylogenetically closely related species do not appear to align well, suggesting that the pattern of the urmylation of Urm1 is not conserved. Furthermore, it remains to be determined if the formation of poly-urmylation chains correlates with different substrate fates.

A large number of intracellular proteins were urmylated. Treatment with the proteasome inhibitor bortezomib increased the overall intensity of Urm1 conjugates and altered the structure of poly-urmylation chains, strongly suggesting that urmylation serves as a signal for proteasomal degradation. In this study, 46 ribosomal proteins were found to be urmylated at one to nine lysine residues. In comparison, approximately 30 ribosomal proteins are ubiquitinated in yeast (9, 56). As a quality control mechanism, ubiquitination contributes to the degradation of ribosomal proteins, facilitating the clearance of excess free ribosomal proteins to maintain the stoichiometric production of all ribosomal proteins for the optimal function of ribosomes (57–59). Furthermore, ubiquitination of ribosomal proteins would mediate the turnover of mature ribosomes under conditions of cellular stress or upon ribosome collisions caused by translational stalling or the formation of non-functional rRNA-containing ribosomes (59–61). Urmylation of ribosomal proteins likely serves similar roles in *S. islandicus*.

Urmylation was influenced by growth conditions and stress treatments. For example, treatment with the oxidant diamide induced the conjugation of free Urm1 to substrate proteins. A similar observation was made on eukaryotic Urm1 (25, 28) and SAMPs from *H. volcanii* (47, 48), suggesting that the response of Urm1 homologs to oxidative stress is conserved in both Eukarya and Archaea. It was previously shown that the peroxiredoxin Ahp1, a typical substrate of eukaryotic Urm1, was involved in oxidative stress response (20, 21, 27, 62, 63). Ahp1 was urmylated at K32 and K156, and a significant increase in Ahp1-Urm1 conjugates, with a molecular mass of ~40 kDa, was observed following treatment with diamide, but not with other oxidants such as t-BOOH, in yeast (27, 62). Interestingly, two heavily urmylated proteins with molecular masses of ~40 kDa were detected by Western blotting in *S. islandicus* following diamide treatment (Fig. 5C; Fig. S7). These two proteins remain to be identified, but it is tempting to speculate that they are the *S. islandicus* homologs of eukaryotic Ahp1, since two peroxiredoxin proteins (SiRe_0067 and SiRe_2592) from the strain were found to be urmylated at several sites in this study. If they were modified simultaneously with two Urm1 molecules, the resulting conjugates would be approximately 40 kDa in molecular mass.

Urm1, the sole Ubl homolog in *S. islandicus*, exhibits some characteristics typical of ubiquitin, including the ability to urmylate a large number of substrates and to catalyze poly-urmylation (3, 5, 8–10), but also resembles eukaryotic Urm1 in structure and in possessing a single activating enzyme, i.e., Uba4p, and being synthesized as a mature protein (17, 39). These findings are consistent with the hypothesis that eukaryotic Ub/Ubl systems have an archaeal origin (30). Moreover, it is highly likely that *S. islandicus* Urm1 may mediate proteolysis in a similar fashion to eukaryotic ubiquitin.

Our attempts to construct an *S. islandicus* mutant lacking Urm1 were unsuccessful, suggesting that *urm1* is an essential gene. We found that the growth of *S. islandicus* was closely linked to the availability of free Urm1 in the cells, with rapid growth occurring only when sufficient free Urm1 was present. The cellular content of free Urm1 depended strongly on the growth phase, being highly abundant until the death phase, during which the free protein was no longer detectable. Following inoculation into the induction medium, the *urm1* knockdown strain *urm1-kd*, in which little free Urm1 existed, exhibited delayed growth and started to show apparent growth only when free Urm1 accumulated in the cells. It is worth noting that the reduced levels of free Ub diminish the viability of yeast and mice (15, 16).

Significantly, the period of delayed growth coincided with the drastic decrease in the cellular levels of the cell division proteins (CdvB, CdvB1, and CdvB2) in the *urm1-kd* strain. Inactive cell division as the result of the lowered levels of cell division protein is likely a mechanism underlying the delayed growth. It has been shown in *S. acidocaldarius* that a CdvB ring forms first at the middle of the cell, and CdvB1 and CdvB2 are subsequently recruited to form a division ring (64). Degradation of CdvB, mediated by the proteasome, triggers cell division by facilitating the constriction of the division ring. The increase in Cdv protein contents is transcriptionally regulated (65, 66), but the mechanism of Cdv protein degradation is less clear. Since CdvA, CdvB1, and CdvB2 were found to be urmylated, it is tempting to suggest that the modification is involved in the control of cell division by serving as a signal for the proteasomal degradation of the cell division proteins. However, this suggestion is not supported by the observed downregulation of CdvB1 and CdvB2 in the *urm1-kd* strain. It is more likely that the reduction of the cellular Urm1 levels affected a wide range of cellular activities, resulting indirectly in the decrease in the cellular amounts of CdvB1 and CdvB2. It was shown that the knockdown of *cdvABC* resulted in cell enlargement and the increase in DNA content in the cell (51). In this study, however, *urm1-kd* cells, which possessed downregulated CdvA, CdvB1, and CdvB2, were smaller than the control cells. Therefore, it appears that the general impact of the *urm1* knockdown on cell physiology hinders not only cell division but also cell growth.

## Data Availability

The mass spectrometry proteomics data have been deposited to the ProteomeXchange Consortium via the PRIDE (67) partner repository with the datasetdata set identifier PXD057602 (https://proteomecentral.proteomexchange.org/).

## SUPPLEMENTAL MATERIAL

Supplemental material msystems.00580-25-s0001.docx Fig. S1 to S9; Table S1.

Table S2 msystems.00580-25-s0002.xlsx A list of the urmylated peptides identified.

Table S3 msystems.00580-25-s0003.xlsx All peptides of linkage sites.

Table S4 msystems.00580-25-s0004.xlsx Differentially regulated proteins in *urm1* knockdown strain.

ASM does not own the copyrights to Supplemental Material that may be linked to, or accessed through, an article. The authors have granted ASM a non-exclusive, world-wide license to publish the Supplemental Material files. Please contact the corresponding author directly for reuse.
