# Supplementary material for: Protein modification by a eukaryotic-like ubiquitin-related modifier in the hyperthermophilic archaeon Saccharolobus islandicus
Source: mSystems. 2025 Oct 20;10(11):e00580-25. doi: 10.1128/msystems.00580-25 (PMC12625751; doi:10.1128/msystems.00580-25)
Supplement: Supplemental material — Fig. S1 to S9; Table S1. [file msystems.00580-25-s0001.docx]

**Supplementary materials**

**Protein modification by a eukaryotic-like ubiquitin-related modifier in the hyperthermophilic archaeon *Saccharolobus islandicus***

Jingjing Cao, Daijiang Xiong, Xiaowei Zheng, Wanjuan Yuan, Li Huang

The file includes Table S1 and Figures S1 to S9.

Tables S2, S3, and S4 are provided as separate individual Excel files.

Table S1. Primers used in this study.

| Name^a^ | Sequence^b^ | | Use |
| --- | --- | --- | --- |
| Spacer-fwd | AAAGTAAATCATGGTGGTTAGAGATATCGAAGGTTCAAGTAAGT | | Construction of *urm1* H81R strain |
| Spacer-rev | TAGCACTTACTTGAACCTTCGATATCTCTAACCACCATGATTTA | |  |
| dornor-fwd | ACCCGTCGACGAGGTAAGGAGAACAGCTG | |  |
| dornor-rev | ATAGCGGCCGCGGTACCATGAAAAACACTAAG | |  |
| Flanking-fwd | CCAGTTATGGTAGTAAGAGCTC | |  |
| Flanking-rev | CTTCAAACGGTACGATAGG | |  |
| Pet30a-dornor-fwd | GAAGGAGATATACATATGGAGGTAAGGAGAACAGCTG | |  |
| Pet30a-dornor-rev | GTGGTGGTGGTGCTCGAGGGTACCATGAAAAACACTAAG | |  |
| dornor-Mut1-fwd | AGATAATACCgATAAATCATGGTGGTTAGAGATATCGAAGG | |  |
| dornor-Mut1-rev | CATGATTTATcGGTATTATCTCAATGATATCATTTTCATTTAAAAATTGATC | |  |
| dornor-Mut2-fwd | ACCGATAAATagaGGTGGTTAGAGATATCGAAGGTTCAAG | |  |
| dornor-Mut2-rev | TCTAACCACCtctATTTATCGGTATTATCTCAATGATATCATTTTC | |  |
| Spacer-fwd | AAAGTTCTTTATCACTGACGTCTATTTCTCTGCAATTAAGTTGA | Contruction of *urm1-knockdown* strain | |
| Spacer-rev | TAGCTCAACTTAATTGCAGAGAAATAGACGTCAGTGATAAAGAA |  |  |
| Screening-fwd | AACTGGCGGTACATAGTGGTA |  |  |
| Screening-rev | GGGTAGAAGTGTGTATGAG |  |  |
| Urm1-fwd | GAAGGAGATATACATATGCCGAAAGTGATATTAAAGG | Recombiant expression | |
| Urm1-rev | GTGGTGGTGGTGCTCGAGCTAACCACCATGATTTATGGG |  |  |

^a^ fwd, forward primers; rev, reverse primers; Mut, mutant.

^b^ Restriction sites are underlined. Lowercase letters indicate bases to be introduced to replace those in a wild-type gene.

Figure S1


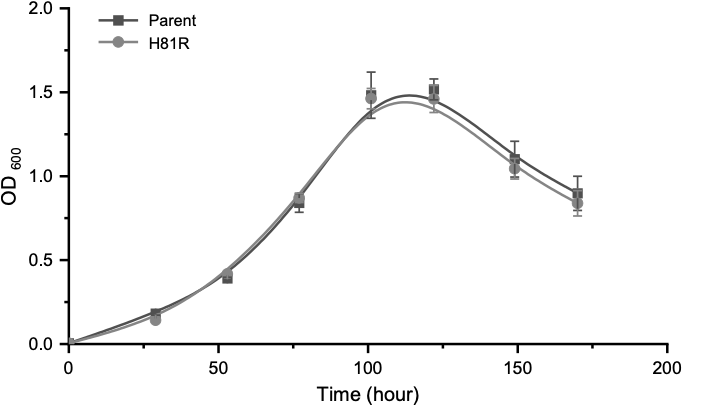


Figure S1. Growth curve of the H81R mutant strain. The H81R and the parent strains were incubated in SCVy medium. All data points are an average of three independent measurements.

Figure S2


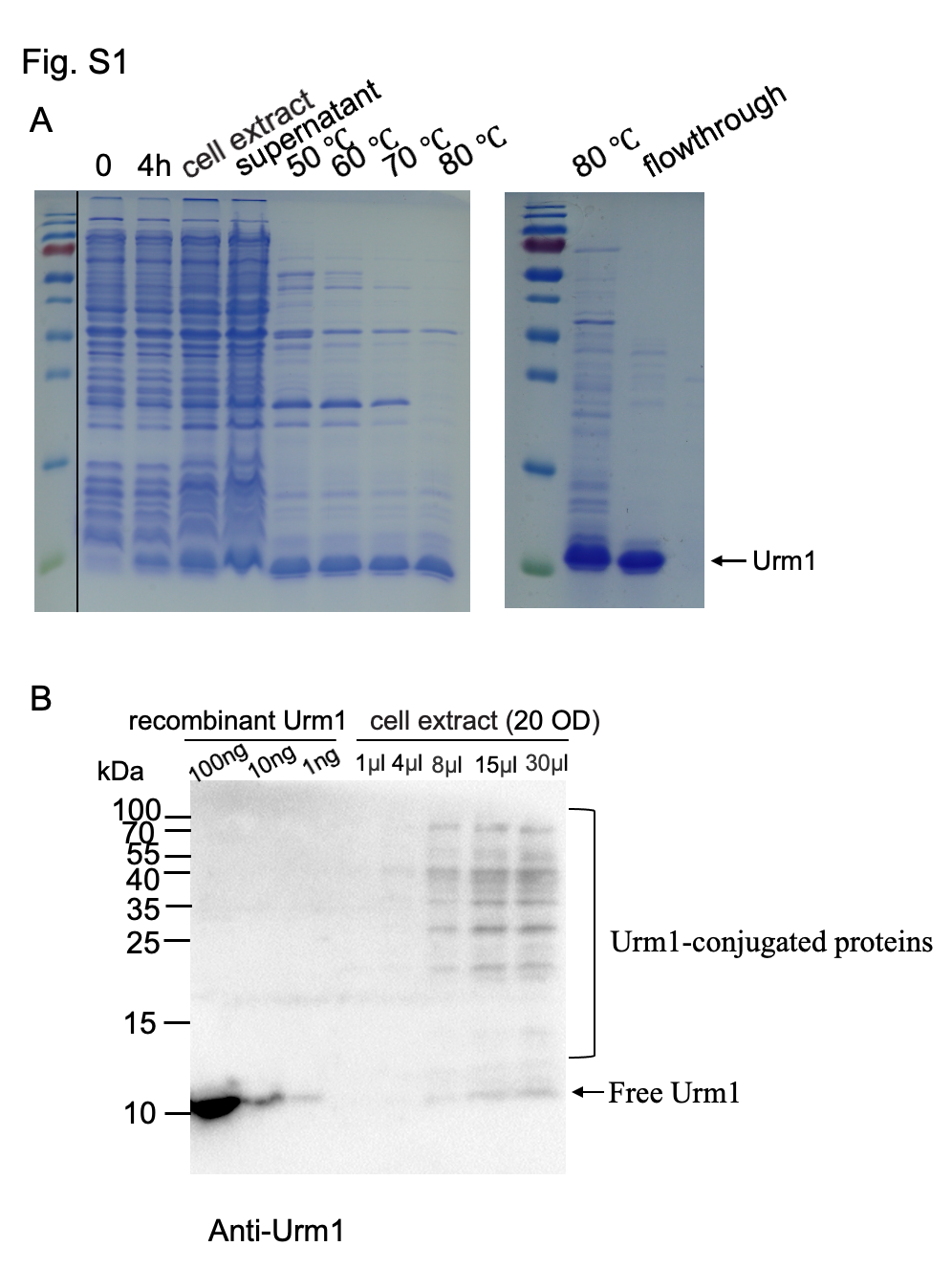


Figure S2. The preparation of the antibody against Urm1. (A) The purification of the recombinant Urm1. Urm1 genes (SiRe_1713) were cloned into expression vector pET30a by SiRe1713-forward primer (GAAGGAGATATACATATGCCGAAAGTGATATTAAAGG) and SiRe1713-reverse primer (GTGGTGGTGGTGCTCGAGCTAACCACCATGATTTATGGG). The resultant expression vector was transformed into *E. coli* Rosetta (DE3). The recombinant Urm1 was induced with the addition of 0.8 mM isopropyl-1-thio-β-D-galactopyranoside (IPTG) for 4 h. Then, the cells were harvested by centrifugation, resuspended in buffer A [20 mM Tris-HCl, pH 8.8, 1 mM DTT, 0.1 mM EDTA, 500 mM NaCl, 10% (vol/vol) glycerol] and sonicated on ice. After centrifugation, the supernatant was heat treated at 80°C for 20 min. Samples were clarified by centrifugation at 30,000 x g for 30 min at 4°C. The sample was loaded onto a 1 ml HiTrap Q column and the flowthrough were collected for development of the antibody. (B) The specificity of the anti-Urm1 antibody was assessed by immunoblotting. A series of the recombinant Urm1 and the *S.islandicus* cell extract at different amounts were measured by immunoblotting using anti-Urm1 antibody.

Figure S3


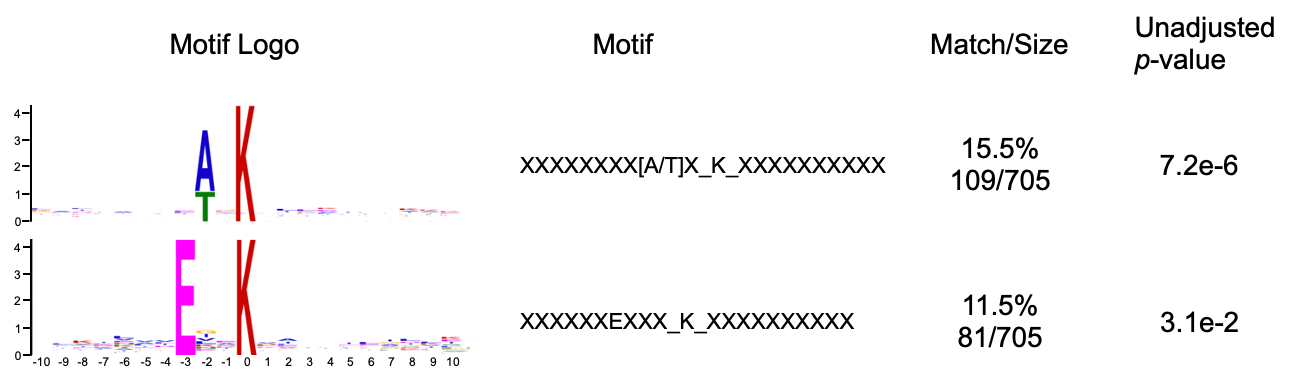


Figure S3. Motif analysis of urmylation peptides. Two conserved amino acid sequence motifs spanning from position −10 to +10 with respect to the modified lysine residue were identified from 705 peptides. Urmylated lysine motifs were analyzed by MoMo modificaton motifs (https://meme-suite.org/meme/tools/momo). The height of each letter represents the frequency of that amino acid residue in that position. The central K is the urmylated lysines. Match/Size was the ratio of the number of peptides that match the motif to the total number of peptides. Significantly enriched motifs were identified with cutoffs set at p-value < 0.05.

Figure S4


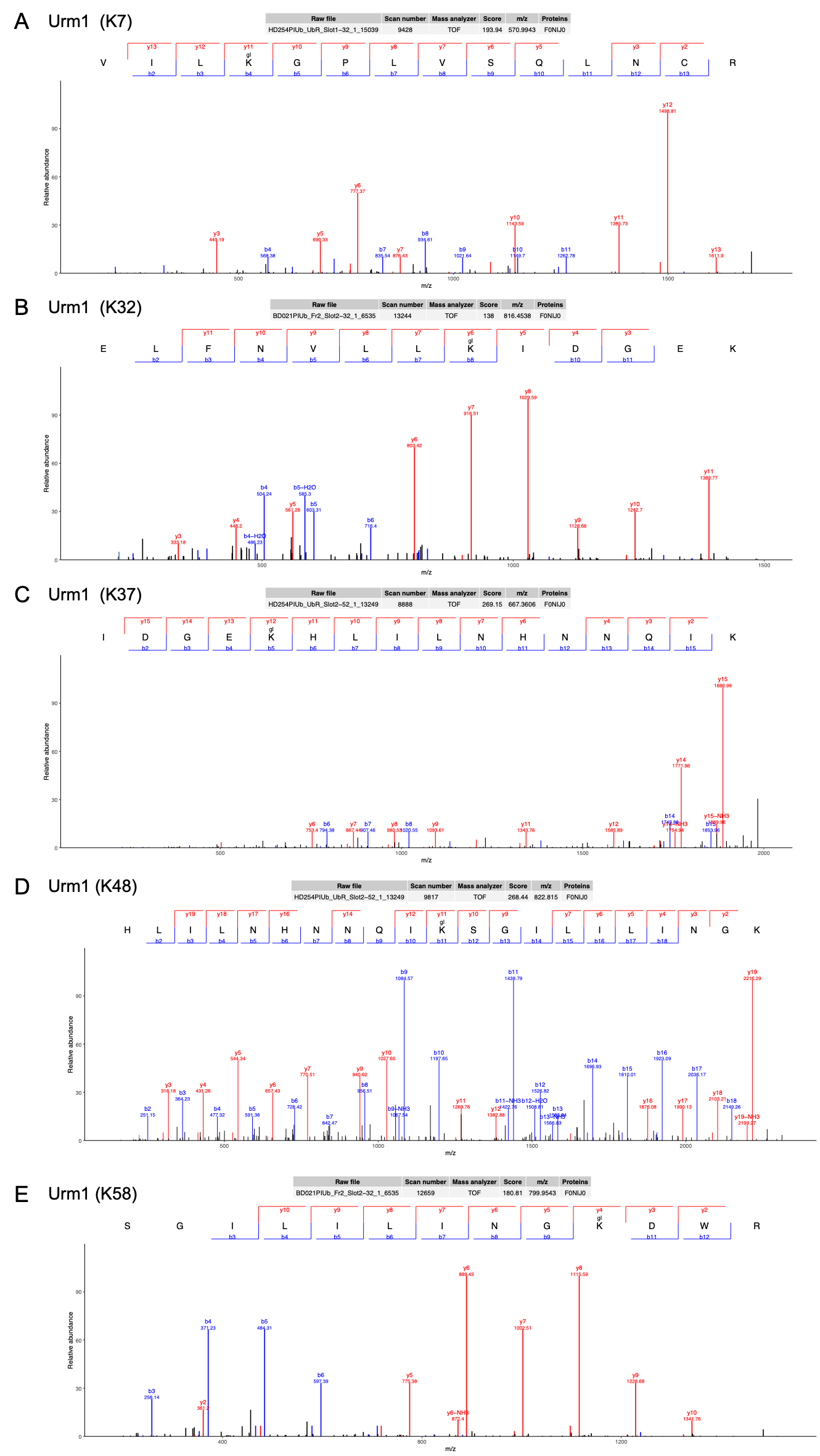


Figure S4. The MS^2^ spectra of the peptides indicated Urm1 modified itself at K7, K32, K37, K48 and K58. The m/z values of the precursor ions are indicated in the top right of each panel. Spectra show the annotated peaks that are due to C-terminal y (colored red) and N-terminal b (colored blue) fragment ions. The m/z values of the precursor ions and the values of the fragment ions revealed that Urm1 were modified with di-glycine at K7, K32, K37, K48 and K58. gl, di-glycine.

Figure S5


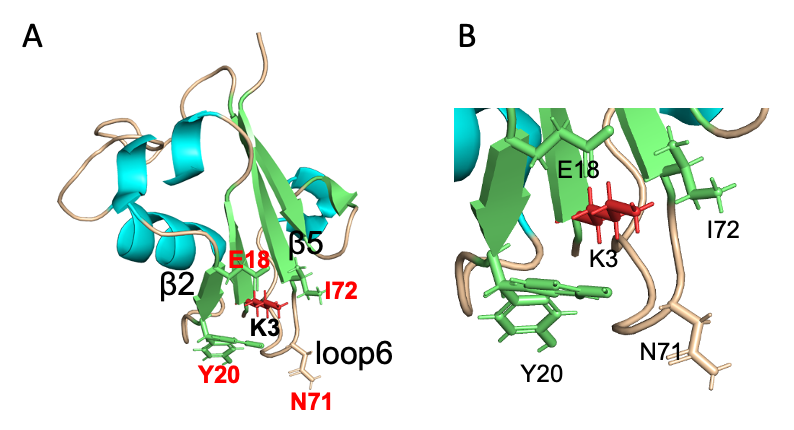


Figure S5. Spatial structure of K3 in Urm1 structure. 3D ribbon models of *S. islandicus* Urm1 highlighting the K3. Adapted from *S. solfataricus*Urm1 structure (PDB: 4WWM). α helices, β strands, and loops are shown in cyan, green, and tan, respectively. Side chain of K3was marked by red, the E18, Y20 from β2, I72 from β5 side chains were marked by green, and N71 side chain from loop6 was marked by tan. (B) An enlarged view of the interfaces of K3 and other residues.

Figure S6


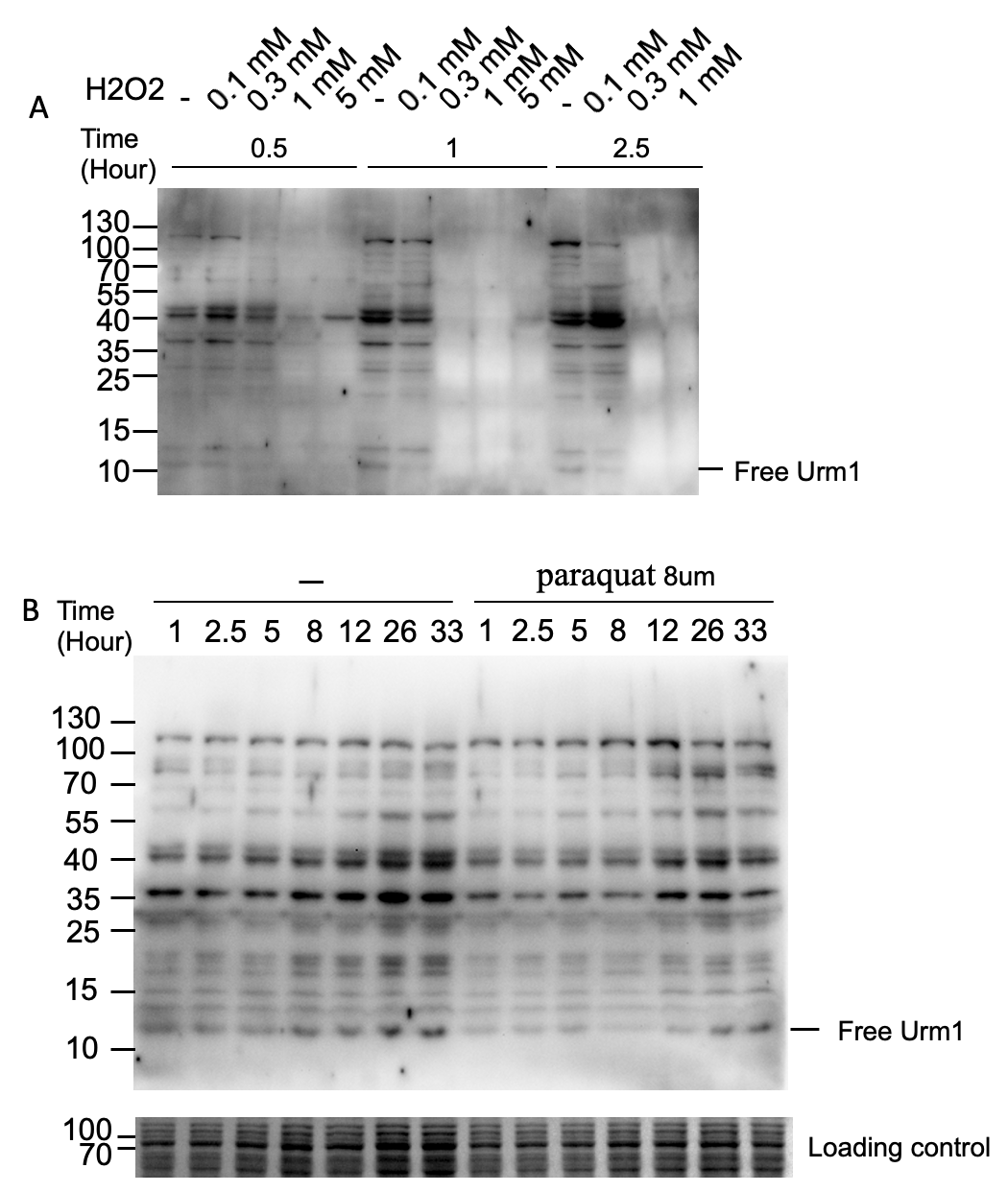


Figure. S6. Effect of oxidants H_2_O_2_ or paraquat on urmylation. (A) The cells during logarithmic stage were treated by 0.1 mM, 0.3 mM, 1mM, and 5mM H_2_O_2_ or ddH_2_O (control). Samples were collected after treatment 0.5, 1.0, or 2.5 hours. Then samples were resolved by SDS-PAGE, and subjected to anti-Urm1 immunoblotting. (B) The cells during logarithmic stage were treated by 8 μM paraquat. Samples were collected for treatment 1, 2.5, 5, 8, 12, 26, 33 hours, then were resolved by SDS-PAGE, subjected to anti-Urm1 immunoblotting. The same sample were subjected to SDS-PAGE and stained with Coomassie brilliant blue G250, serving as a loading control (lower panel). H_2_O_2_ and paraquat did not significantly altered the global protein urmylation. When the concentration was above 1 mm, H_2_O_2_ induced the cell lysis.

Figure S7


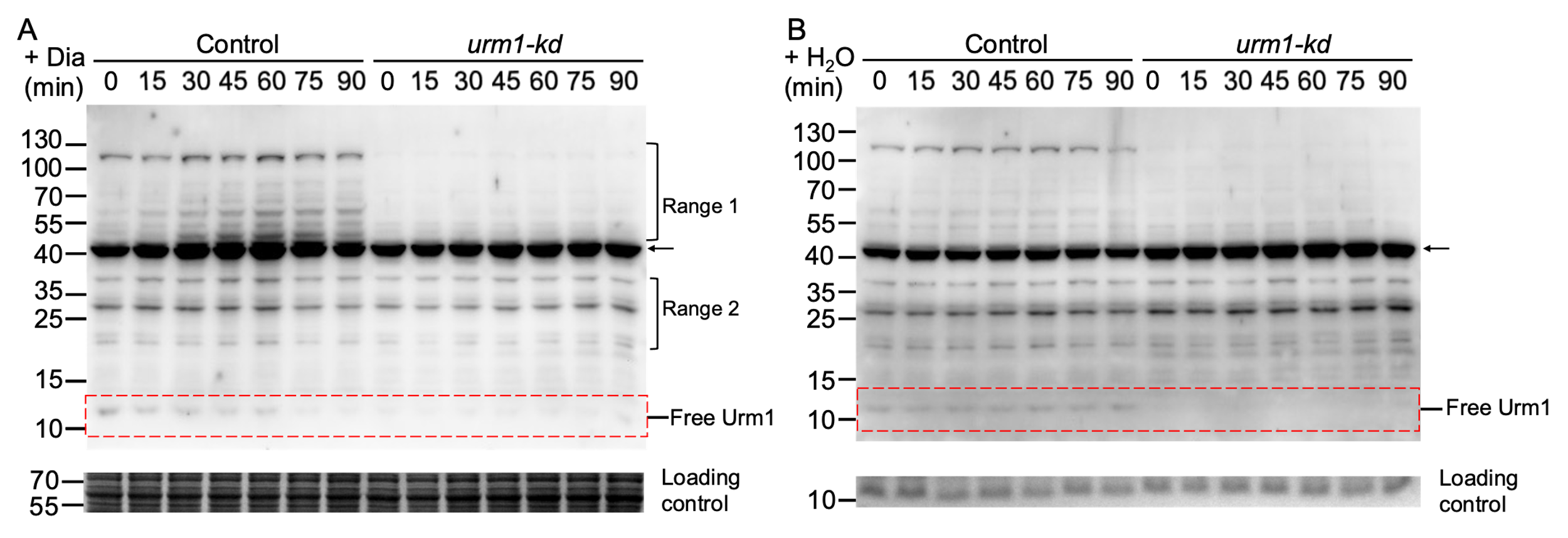


Figure. S7. Effects of diamide on urmylation over time. The control strain and the *urm1*-*kd* strain were cultured in ACVy medium in which sucrose was replaced by arabinose in order to induce *urm1* silencing. The control and the *urm1*-*kd* cells during logarithmic stage were treated by 1 mM diamide (A) or ddH_2_O (B) for 90 minutes. Samples were collected every 15 minutes. Then equal amounts of proteins of each sample were resolved by SDS-PAGE, and subjected to immunoblotting using anti-Urm1 antibody. The loading control was prepared as described above. In A and B, the strongest ladder marked by a black arrow was an unidentified protein induced by arabinose. In panel A, the higher and lower molecular-weight protein ladders were marked as rang 1 or rang 2, respectively. Free Urm1 was marked by red box.

Free Urm1 was barely detectable in the *urm1-kd* strain and was significantly lower than in the control strain. Notably, in the control strain, the intensity of conjugate ladders in range 1 progressively increased following diamide treatment, peaking at 60 minutes and then gradually decreasing (A). The intensity of conjugate ladders in range 2 showed no significant changes over the treatment duration. As diamide treatment continued, the amount of free Urm1 in the control strain decreased over time, becoming nearly undetectable after 60 minutes, comparable to the levels of free Urm1 in *urm1-kd* strain. In *urm1*-*kd* strain, the lack of sufficient free Urm1 prevented any significant changes in Urm1 conjugates in response to diamide treatment. Without diamide treatment, the overall levels of Urm1 conjugates remained unchanged over time in both the control and *urm1-kd* strain (B).

Figure S8

Figure S8. Relative expression level of *urm1* in *urm1-kd* strain compared to the control strain. Total RNAs of the control and *urm1-kd* strains, when both strains OD_600_ were about 0.3, were extracted with TRIzol (Invitrogen) according to the manufacturer’s instruction, respectively. After DNaseI digestion, total RNA was reverse transcribed into cDNA using M-MLV Reverse Transcriptase (Promega, Fitchburg, WI), as described previously. Primers (forward: TGCCGAAAGTGATATTAAAGGG, reverse: CTCGCCATCTATCTTCAGTAATAC) were designed using Primer Premier 6.0. Quantitative PCR (qPCR) reaction mixtures contained 2× KAPA SYBR® FAST qPCR Master Mix Universal (10 μl; KAPA Biosystems, Wilmington, DE), 50-fold diluted cDNA (1 μl), and 200 nm primers in a final volume of 20 μl. qPCR reactions were conducted on a LightCycler 480 II PCR machine (Roche, Basel, Switzerland) according to the manufacturer's protocol. Relative mRNA expression was calculated using the comparative threshold cycle (Ct) method. The level of 16S rRNA was used as a reference to normalize the expression data for target genes.

Figure S9


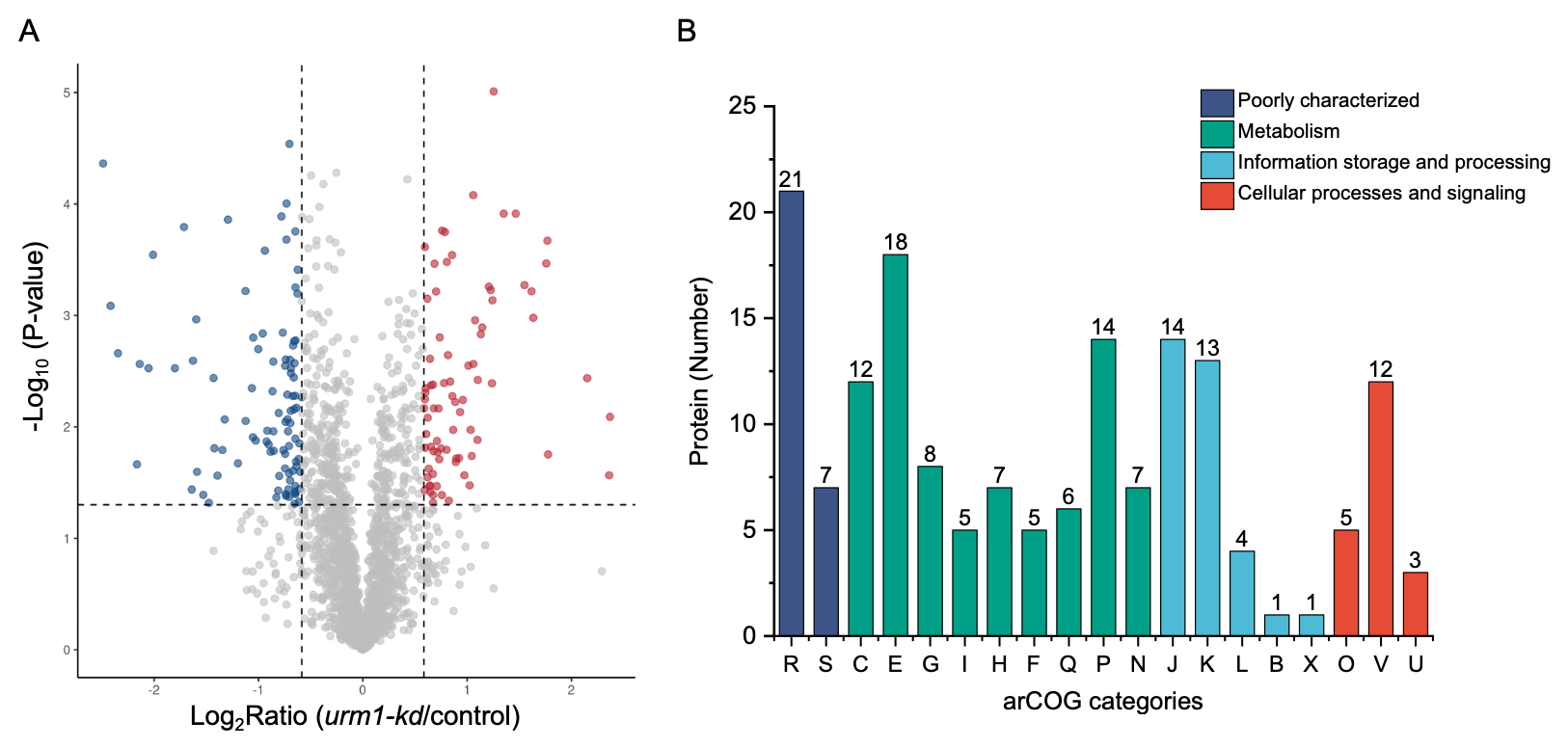


Figure. S9. Analysis of the differentially regulated proteins in *urm1-kd*. (A) A volcano plot showing proteins quantified in the proteomic analysis of the *urm1-kd* and the control strains. Differentially regulated proteins were identified with cutoffs set at p-value ≤0.05 and fold-change ratio ≥1.5 fold. Significantly down- and upregulated proteins are shown in blue and red, respectively. (B) arCOG analysis of the differentially regulated proteins in *urm1-kd*. R, general function prediction only; S, function unknown; C, energy production and conversion; E, amino acid transport and metabolism; G, carbohydrate transport and metabolism; I, lipid transport and metabolism; H, coenzyme transport and metabolism; F, nucleotide transport and metabolism; Q, Secondary metabolites biosynthesis, transport, and catabolism; P, inorganic ion transport and metabolism; J, translation, ribosomal structure and biogenesis; K, transcription; L, replication, recombination and repair; B, chromatin structure and dynamics; O, post-translational modification, protein turnover, and chaperones; V, defense mechanisms; N, cell motility; X, mobilome: prophages, transposons; U, intracellular trafficking, secretion, and vesicular transport.

Method: The *urm1-kd* and control strains were prepared in triplicate for label-free quantitative proteomic analysis. Cells were harvested at an OD600 of 0.3 and subsequently subjected to ultrasonic treatment on ice. Equal amounts of proteins from each sample were alkylated, precipitated with acetone, dissolved in DB buffer, and digested with trypsin. The resulting tryptic peptides were analyzed using a Vanquish Neo upgraded UHPLC system coupled with a Thermo Orbitrap Astral mass spectrometer. The primary MS resolution was set to 240,000 (200 m/z), AGC was set to 500%, the parent ion window size was set to 2-Th, and 300 DIA windows were used. The NCE was set to 25%, the secondary m/z acquisition range was 150-2000, and the sub-ion resolution was set to 80,000, with a maximum injection time of 3 ms. The raw files were analyzed using DIA-NN search software (DirectDIA) based on the Uniprot *S. islandicus* REY15A database (2018/12/26, taxonomy ID: 930945, 2,631 sequences).
